# Longitudinal craze line propagation in human root dentin after instrumentation with NiTi rotary files of different instrument tapers after long-term chewing simulation

**DOI:** 10.1007/s00784-021-04238-3

**Published:** 2021-11-17

**Authors:** Marie-Therese Heberer, Hubert C. Roggendorf, Franz-Josef Faber, Nicolai-Alexander Lawrenz, Roland Frankenberger, Matthias J. Roggendorf

**Affiliations:** 1 Department of Operative Dentistry, Endodontics, and Pediatric Dentistry, Philipps University Marburg and University Hospital Giessen and Marburg, Campus Marburg, Georg-Voigt-Straße 3, 35039 Marburg, Germany; 2 Interdisciplinary Department of Oral Surgery an Implantology, University of Cologne, Kerpener Straße 32, 50931 Cologne, Germany; 3 Pre-Clinical Department, University of Cologne, Kerpener Straße 32, 50931 Cologne, Germany; 4 Department of Mathematics and Computer Science, Philipps University of Marburg, Hans-Meerwein-Straße 6, 35043 Marburg, Germany

**Keywords:** Craze lines, Microcracks, Taper, Cutting-edge angle, Digital microscopy, Root dentin

## Abstract

**Objectives:**

The aim of this study was to investigate whether file design and taper significantly influence microcrack initiation during machine preparation.

**Materials and methods:**

Sixty extracted teeth with straight single canals were selected. The teeth were randomly assigned to four groups based on their root canal anatomy and the corresponding NiTi rotary file system (I, *Mtwo*; II, *ProTaper Universal*; III, *F6 SkyTaper*; control, no preparation and filling). The root canals of the experimental groups were filled using the single-cone technique. The tested teeth were all subjected to a mechanical chewing simulation with flat lead loading over a period of 3 years (corresponding to 150,000 cycles). The teeth were checked for dentinal defects (accumulative crack growth in length) under the digital microscope (*Keyence VHX-5000*) at time 0 (baseline prior to chewing simulation) and after 3, 6, 12, 24, and 36 months of loading. The cumulative crack increase was statistically analyzed using the Kruskal–Wallis test, Jonckheere–Terpstra test, and the Wilcoxon rank-sum test. The significance was set at *p* < 0.05.

**Results:**

In contrast to preparation with greater-tapered instruments, *ProTaper Universal* (group II) and *F6 SkyTaper* (group III) instrumentation with the smaller tapered *Mtwo* files (group I) showed less accumulative propagation of craze lines (*p* < 0.05) at all time points.

**Conclusion:**

Instruments with greater taper for root canal instrumentation should be used with care to avoid negative long-term effects in the form of propagation of dentinal defects over time. A positive cutting-edge angle and a smaller taper have a positive effect on a lower craze line development.

**Clinical relevance:**

Instruments with a positive cutting-edge angle and a smaller taper are beneficial for the long-term preservation of dentinal tooth structure.

## Introduction

Microcracks are defined as fine, incomplete craze lines that occur in areas of force concentration if the elastic limit of the tooth structure is exceeded, so they appear in most adults [1]. The propagation of microcracks may result in vertical root fractures (VRFs) which can potentially be a reason for extraction of the affected tooth [2–5]. Reasons for defects in dentine can be iatrogenic factors in the course of endodontic treatment, such as the opening of the tooth through a central access cavity, instrumentation [6, 7], the irrigation protocol used [8, 9], the medicinal insertion [10], or the filling technique [11–13]. When instrumenting a root canal, the canal wall is always exposed to forces by instrumentation and may be damaged, which can potentially result in the propagation to incomplete or complete fractures [13, 14]. The instrument design and the number of files used have been shown to affect the probability of microcracks developing in the radicular dentin [5, 6, 15–17]. However, the influence of endodontic treatment on microcrack formation is highly controversial. Some authors assume that the microcracks observed in the aforementioned studies are much more a consequence of the extraction process in in vitro studies [18, 19]. The microcrack formation is said to be independent of the extraction technique but a consequence of the dehydration process outside the oral cavity [19, 20]. In this way, the methodology of the earlier mentioned studies is questioned [19].

In this study, three different instrument systems were used. *Mtwo* (VDW GmbH, Munich, Germany) was the first system developed for fully-rotating instrumentation of root canals using the single-length technique. An advantage here is the guiding function of the non-cutting instrument tip for good centering in particularly curved canals. As soon as one instrument reaches full working length, the operator can switch to the next file size [14, 21–24]. The *ProTaper Universal* system (Dentsply Sirona GmbH, Bensheim, Germany) combines different tapers within one instrument as expressed by the name (Pro = progressive, Taper = instrument taper). After preparation of the access cavity, the canal entrance is enlarged by the shaping files SX, S1, and S2, followed by the instrumentation of the apical canal portions with finishing files from F1 to F4 depending on the root canal anatomy. The instrumentation is performed using a crown-down preparation [14, 25]. The third system used in this study is the single-file system *F6 SkyTaper* (Komet Dental GmbH & Co. KG, Lemgo, Germany). The manufacturer advertises the high flexibility of these files. The canal preparation should be performed using three in-and-out movements, cleaning of the instruments, followed by intermittent irrigation of the root canal [26, 27]. Instrumentation is finished when the instrument reaches working length without binding to the canal wall.

The present study investigates whether craze line propagation can be related to endodontic treatment. For this purpose, the methodology was checked in advance using in a preliminary test. If a relation can be shown, the influence of instrument taper, cutting-edge angle, number of files, and design (reverse vs. continuous taper) on the formation and increase of craze lines in root dentin will be investigated. The null hypothesis was that the instrument design has no significant influence on craze line formation.

## Materials and methods

Prior to the start of the main experiment, a pre-group (*n* = 15) was selected and pre-treated in the same way for a preliminary proof to confirm the methodology used. The aim was to analyze the effect of repeated drying on crack development. For this purpose, twelve digital microscope images were taken of each tooth, and the crack increase was analyzed. The teeth were only dried for the exposures and otherwise stored wet.

The sample size was calculated a priori using the software *R* (R Foundation for Statistical Computing, Vienna, Austria) performed by a chi-square power test (with α = 0.05, β = 0.95) [28]. A predefined power of 0.9 resulted in a sample size of 15 teeth per group as the required target value. Therefore, we used a total sample size of 60 teeth (*n* = 15).

**Fig. 1:**
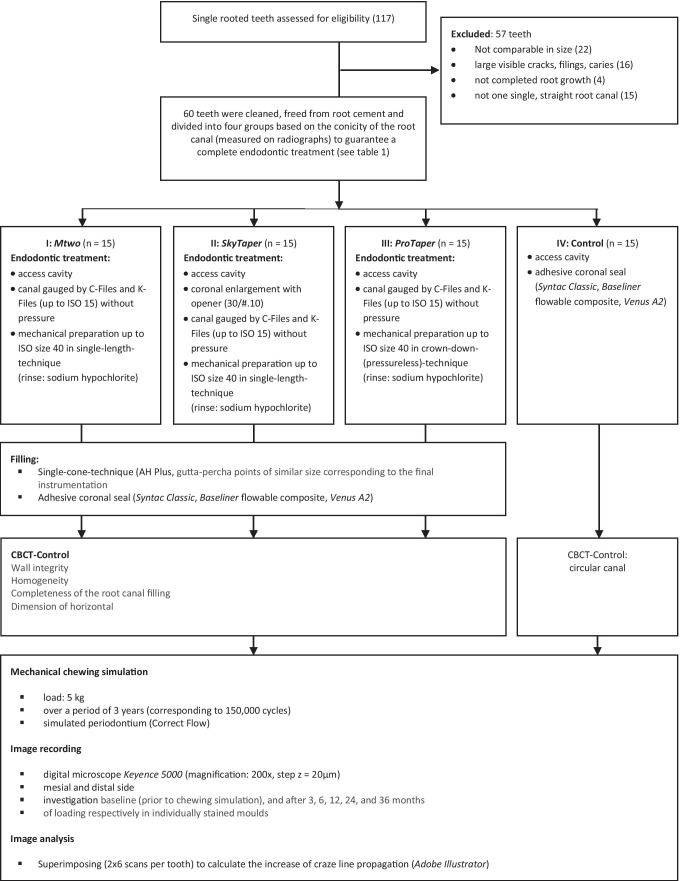
The experimental procedure of the main trial

Figure 1 shows the experimental procedure of the main trial. Throughout the study, all teeth were stored in 0.9% isotonic NaCl solution with 0.001% sodium azide. The root canal preparation of the different experimental groups (see Table 1 and Fig. 1) was carried out with the torque-limited endomotor *EndoPilot1* (Schlumbohm GmbH & Co. KG, Brokstedt, Germany). All instruments were used according to the manufacturer instruction. The simulation of periodontal tissue was performed by the use of the thin-flowing type A-silicone *Correct flow* (Kulzer GmbH, Hanau, Germany) as it was realized in several other studies [35]. During instrumentation of the teeth, the root canals were filled with NaOCl 3%, and irrigation was unable to disappear due to the embedding in the molds.

**Table 1:** Overview of the experimental groups [17, 27, 29–34]

Group	File system	File sequences	Manufacturer	Taper	Cross-section	Technique
I	*Mtwo*	#10/.04, #15/.05, #20/.06, #25/.05, #30/.06, #35/.04, #40/.04	VDW Dental, Munich, Germany	Continuous taper .04	S-shaped (active cutting) with a large chip space and small core diameter	Single-length (full rotation)
II	*SkyTaper F6*	Opener #30/.10, #40/.06	Komet Dental, Lemgo, Germany	Continuous taper .06	Double-S-shaped (active cutting) with a large chip space and small core diameter	Single-length (full rotation)
III	*ProTaper Universal F4*	SX, S1, S2, F1 (#20/.07), F2 (#25/.08), F3 (#30/.09), F4 (#40/.06)	Dentsply Sirona, Bensheim, Germany	Reverse taper .06 (different progressive tapers from 2% up to 11.5%)	Up to F3: slightly convex triangular (passive-cutting), F4 concave	Crown-down (pressure-less) technique (full rotation)
IV	Occlusal cavity, composite filling, no preparation and filling		–-	–-	–-	–-
Control	No treatment, no chewing simulation, only digital microscopic scans		–-	–-	–-	–-

**Fig. 2:**
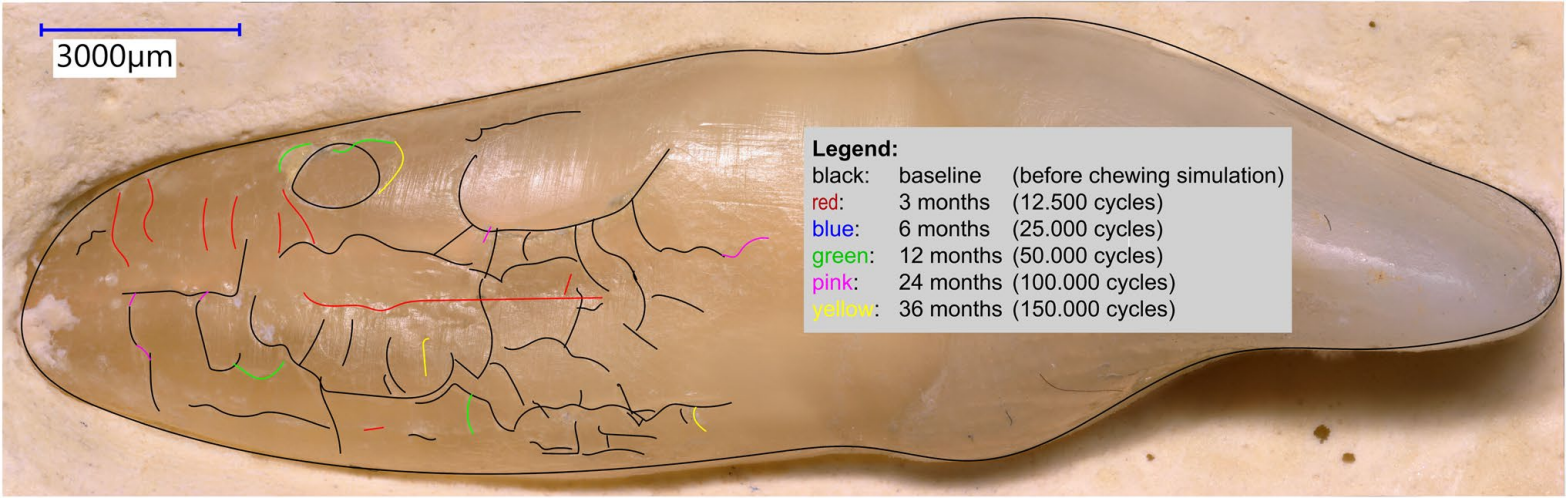
Before starting the chewing simulation (baseline), the teeth were examined for microcracks from both contralateral sides with digital microscopic scans (Keyence VHX-5000, lens: Keyence VH-Z20T, Keyence Corp., Osaka, Japan) (magnification: 200 × , step z = 20 µm). The images were superimposed, and the crack growths were color-coded and measured afterwards

Before starting the chewing simulation (baseline), the teeth were examined for microcracks from both contralateral sides with digital microscopic scans (Keyence VHX-5000, lens: Keyence VH-Z20T, Keyence Corp., Osaka, Japan) (magnification: 200 × , step z = 20 µm). For this purpose, the molds were individually prepared for each tooth so that each scan could be made from exactly the same position mesially and distally. Repeated scans after 12,500 and 25,000 chewing cycles (representing a masticatory load over 3, 6, 12, 24, and 36 months) enabled subsequent software-based measurement of the crack growth in number and length using *Adobe Illustrator* (Adobe Inc., San José, CA, USA)*.* The images were superimposed, and the crack growths were color-coded and measured afterwards (see Fig. 2). In this way, 12 scans per tooth were taken for precise crack analysis to record the crack propagation over the observation period.

The statistical evaluation was also performed with the statistical software *R* (R Foundation for Statistical Computing, Vienna, Austria). First, the Kolmogorov-Smirnov test was used to examine whether the collected data corresponded to a normal distribution. Since there was no normal distribution of the values in this study, the evaluation of the null hypothesis was carried out exclusively with non-parametric test procedures [36]. The Kruskal-Wallis test was computed to calculate if there were any differences in terms of propagation of dentinal defects between the experimental groups. The Jonckheere-Terpstra test and Wilcoxon rank-sum test were used to evaluate the crack increases between the groups [37].

## Results

**Fig. 3:**
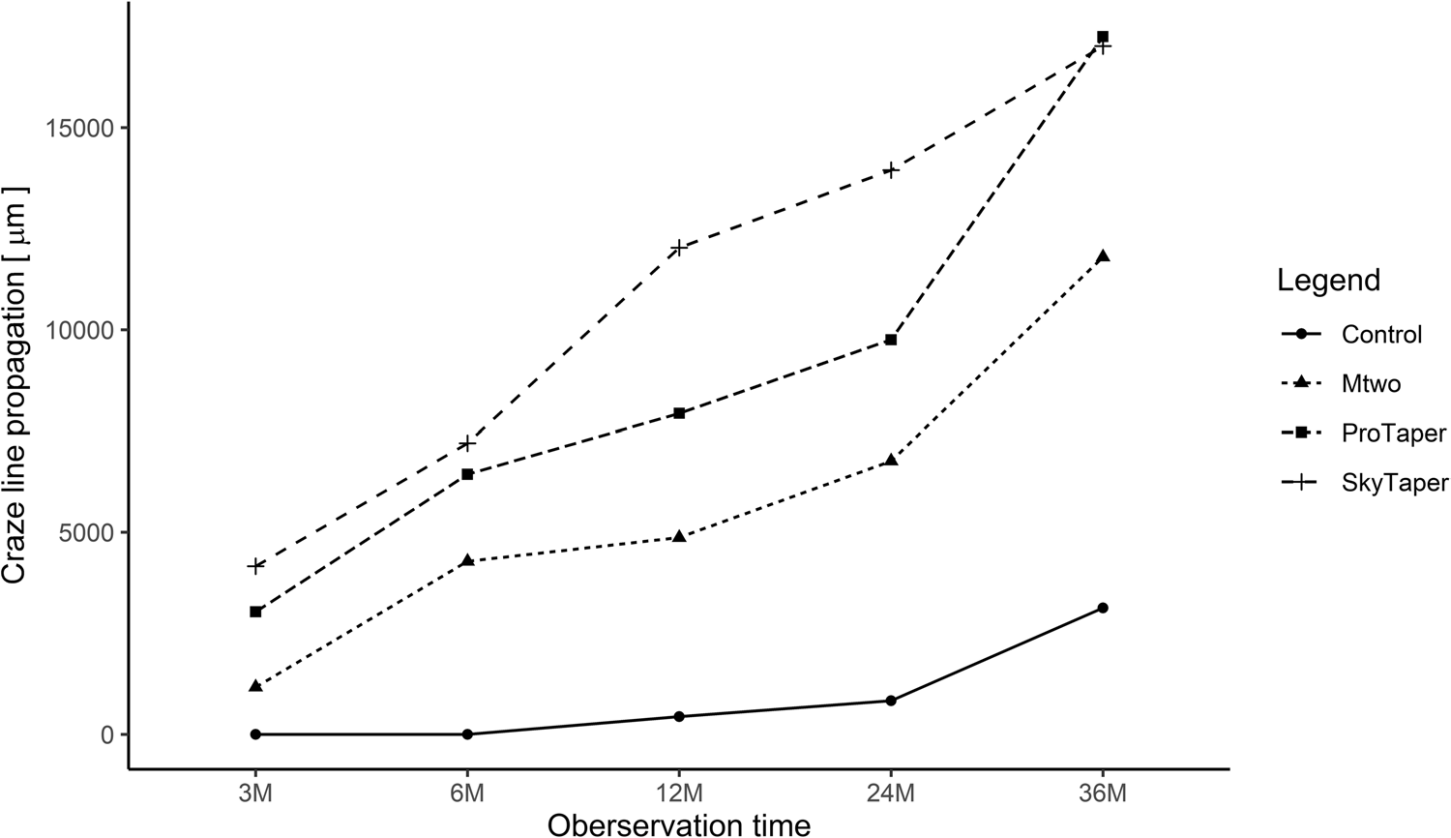
An overview of craze line propagation over the observation period of 3 years starting from baseline [µm]

The pre-trial group did not show any crack increase at any time (0 µm = no accumulative crack increase). Figure 3 gives an overview of craze line propagation over the observation period of 3 years starting from baseline [µm]. The Kruskal-Wallis test confirmed that there were clear differences in crack growth between the groups. The Jonckheere-Terpstra test showed that *Mtwo* led to less cumulative crack growth than the other two groups at all observation points (*p* < 0.5). According to the pairwise comparisons with the Wilcoxon rank-sum test, the results were only significant for the comparison of *Mtwo* with *SkyTaper* up to 24 months (*p* < 0.05). *ProTaper Universal* tended to perform better than *SkyTaper* up to 24 months (*p* < 0.5). However, at 36 months, *ProTaper* showed little higher craze line propagation compared to *SkyTaper*. Even the control group showed a moderate increase between 2 and 3 years.

## Discussion

The current study investigated the occurrence of superficial dentinal craze lines, a type of microcracks that can be detected on the root surface after endodontic therapy. A correlation between the preparation systems used and the increase in craze lines was observed, rejecting the null hypothesis. The methodology used was confirmed by the pre-trial: as there was no propagation of craze lines observed due to the short dry storage mandatory during the scanning process by digital microscopy can be excluded as falsification. As shown by a µCT study of Rödig et al., prolonged dehydration up to 24 h does not result in the generation of new dentinal cracks [38] although this effect was detected by means of a µCT. In the uninstrumented control group (IV, filling only), almost no increase in terms of total craze line propagation was observed (as in other studies) [16, 39, 40]. The slight increase in craze lines was related to the chewing load and the use of human samples. Due to the use of human tooth samples, these were exposed to different stresses and environmental influences before the start of the study, which represents an influencing factor on crack formation. One explanation can be found in natural tooth aging, which leads to changes in the dentin collagen structure as well as a reduced water content and a reduction in bending and fracture strength as well as flexibility [41–44]. Additionally, it should not be neglected that the extraction process also generates forces that may have an impact on the teeth [45]. A correlation for the occurrence of microcracks post extractionem with the patients’ age could also be found [45]. De-Deus’ statement postulated that microcracks must be regarded as a trial-related phenomenon cannot be accepted based on the results of the present study [19, 20, 46]. No VRF occurred in any test group in the present study.

At present, no study has investigated the increase of cracks in freshly treated endodontic teeth in a long-term chewing simulation over several years. For the repeated investigation of the development of craze lines, a digital microscopic analysis of superficial dentinal defects in the form of craze lines was chosen. Optical microscopy of root surfaces and apices has so far only been used to visualize apical crack growth [15, 47–50]. Several studies on dentinal defects after root canal treatment were carried out by microscopy by horizontal sectioning of the roots [6, 13, 14, 35, 39, 40, 51, 52], which have shown to result in an increase in crack formation [53–55]. Although the depth of the cracks can be assessed adequately by this method, no progress control is possible during masticatory loading. However, it was shown that the destructive method of horizontal sectioning resulted in a higher number of dentinal defects when compared with the non-destructive µCT analysis [56]. Digital microscopy is well-suited for imaging microcracks. Depending on the resolution, it is indeed possible that not all present craze lines will be detected [57, 58]. In a study using non-obturated teeth, no significant differences between µCT and stereo-microscopic analysis for the detection of craze lines were detected. The scanning electron microscope detected significantly more craze lines in this analysis, whereas the detectability using CBCT revealed significantly less craze lines [56]. In the present study, electron microscopy was not chosen due to the inability of the SEM technique to allow repeated scans of the specimens. SEM investigation requires a drying process followed by a sputtering of the samples. These pretreatment steps do not allow a repeated chewing simulation. Additionally, repeated vacuuming and increased drying probably would have induced more craze lines [59]. An alternative use of the replica technique for repeated SEM scans would be more than questionable in terms of the reproduction of craze lines because the impression material needs to penetrate cracks in order to allow their detection [60]. Another imaging technique that was repeatedly used in several studies was the µCT. However, this technique was not chosen because the presence of a root canal filling, as classic dental film or CBCT, makes the diagnosis of VRFs more difficult [48]. This can be transferred to the smaller craze lines. Furthermore, Rödig et al. have shown that the moisture content of the specimens is crucial in terms of the detectability of cracks when using the µCT [38]. The results showed that the µCT scans should be performed on dry specimens. Regarding the existing µCT, no information was given on the moisture content of the specimens during the scanning process. Thus, the conclusion that no differences in terms of crack were found in these studies could be questioned. Another aspect that complicates the analysis of small structures such as microcracks in the dentin in µCT images are related to artifacts such as the beam hardening effect caused by obturation materials [61–63]. By using artifact reduction tools such as a copper filter, the beam hardening effect can be reduced, but it cannot be completely eliminated and may lead to a reduction in image quality [61, 64, 65]. Queiroz et al. were able to show that the use of such tools had no influence on the results of the findings, but only subjectively facilitates the analysis [61]. Therefore, we decided to include high magnifications using the digital microscope in order to include color differences and variations in opacity and transparency for a sufficient detection of craze lines.

The results of the main experiment show that smaller tapered instruments represented by the *Mtwo* system caused less craze line propagation than the other two systems at all time points (*p* < 0.05). This is consistent with another paper that compares the craze line incidence of *Mtwo* with *ProTaper Universal* [40]. This can be attributed to the increased cutting performance of *Mtwo* due to its instrument geometry. The triangular cross-section of *ProTaper* results in less space for dentine chips and a smaller cutting efficiency, which, in addition to a lower cleanability [29, 40], also leads to an increase in torque [66, 67]. An additional torque increase occurs due to its greater taper, leading to greater applied vertical force in the apical direction with increasing preparation depth. Thus, additional stress on the dentin and significantly more cracks occur [66, 67]. Significantly more craze line initiation has been detected by using *ProTaper* in high-torque setting [30]. In the present study, a torque-controlled motor was used, which precluded exceeding the maximum torque recommended by the manufacturers. The *EndoPilot1* (Schlumbohm, Brokstedt, Germany) shows good results with regard to its torque limitation [67]. Studies comparing initial instrumentation and retreatment showed that greater manipulation in the apical third of the root canal interior leads to an increase in cracks [6, 52]. In this study, this can be transferred to the comparison of the active cutting system *Mtwo* with its lower wedging effect by the use of the passive-cutting *ProTaper Universal*. The high craze line incidence of *ProTaper Universal* is in line with several other studies [6, 14, 15, 25, 47, 68, 69]. At present, PubMed does not list any studies that compare the incidence of craze lines development of *F6 SkyTaper* (taper.06, constant taper) with one of the other two systems (August 2021). In this investigation, *F6 SkyTaper* performed significantly worse than *Mtwo* (up to 24 months) but similar to *ProTaper Universal*. The explanation can be found in the greater file taper. However, the file cross-section is different and rather comparable to that of *Mtwo* and even performed better in studies regarding cutting efficiency, although not significant [31]. In addition to the good cleaning performance, this can be seen as a positive factor in favor of low craze line formation. However, Pedullà et al. found the instrument geometry in this context to be only a co-factor and place the instrument flexibility due to different alloys at the center of the reasons for the crack increases [17]. As the alloys from the different manufacturers used in the present study were very similar and moreover not thermally modified to reduce their rigidity [17, 25, 70], this statement can be applied to differences in flexibility due to different core diameters. The core diameter increases proportionally with the taper of the instrument. This is another explanation for the lower increase of crack length of the less-tapered *Mtwo* in the comparison with the other two systems. Regarding the fact that the *F6 SkyTaper* is designed as a single-file system, which has the advantage in the reduction of the number of instruments and a shorter working time, those instruments generally revealed a higher incidence of cracking already in other studies [17, 71].

It was surprising that the multifile-system *ProTaper Universal* with a reverse taper did not show superior results in comparison to *F6 SkyTaper* with a constant taper over 36 months. This is in contrast to findings in current literature showing multifile-systems generating lower initial stress and forces applied to the dentinal walls [71–73]. Although a reduction of the load for every single file would be expected, an addition of all files used results in a greater manipulation and potentially a subsequent accumulation of dentinal damage [47]. So this result is quite pleasing for the practitioner due to significant time savings when using a single-file system [29]. Liu et al. compared three single-file systems with *ProTaper Universal* in their 2013 study and found contrasting results [47]. One possible explanation for the comparable results of *F6 SkyTaper* and *ProTaper* in terms of craze line propagation in the present study was that sufficient coronal access was already created by means of the Opener file in favor of the *F6*. In addition, the root canal was gauged using C-files prior to mechanical preparation and a glide paths with ISO size 15 K-files. This step may have optimized the instrumentation resulting in less forces. Another factor in this comparison may be related to the instrument design. *ProTaper Universal* with its triangular cross-section is a passive-cutting system, that shows less cutting efficiency and a smaller chip space for the removal of dentin chips, and consequently leads to stress in the inner root canal walls [29]. In contrast, *F6 SkyTaper* showed very good results in terms of cutting performance [31].

## Conclusion

A positive cutting-edge angle and a smaller instrument taper have a positive effect on the time-dependent craze line development. Microcracks may serve as a precursor of VRFs. Therefore, reduced instrumentation stress may preserve the tooth structure over time.
